# Reversible Dissociation and Ligand-Glutathione Exchange Reaction in Binuclear Cationic Tetranitrosyl Iron Complex with Penicillamine

**DOI:** 10.1155/2014/641407

**Published:** 2014-03-25

**Authors:** Lidia Syrtsova, Natalia Sanina, Konstantin Lyssenko, Evgeniy Kabachkov, Boris Psikha, Natal'ja Shkondina, Olesia Pokidova, Alexander Kotelnikov, Sergey Aldoshin

**Affiliations:** ^1^Institute of Problems of Chemical Physics of the Russian Academy of Sciences, Academician Semenov Avenue Chernogolovka, Moscow Region 142432, Russia; ^2^A.N. Nesmeyanov Institute of Organoelement Compounds of RAS, 28 Vavilov Street, B-334, Moscow 119991, Russia

## Abstract

This paper describes a comparative study of the decomposition of two nitrosyl iron complexes (NICs) with penicillamine thiolic ligands [Fe_2_(SC_5_H_11_NO_2_)_2_(NO)_4_]SO_4_ *·*5H_2_O (**I**) and glutathione- (GSH-) ligands [Fe_2_(SC_10_H_17_N_3_O_6_)_2_(NO)_4_]SO_4_ *·*2H_2_O (**II**), which spontaneously evolve to NO in aqueous medium. NO formation was measured by a sensor electrode and by spectrophotometric methods by measuring the formation of a hemoglobin- (Hb-) NO complex. The NO evolution reaction rate from (**I**)  *k*_1_ = (4.6 ± 0.1)*·*10^−3^ s^−1^ and the elimination rate constant of the penicillamine ligand *k*_2_ = (1.8 ± 0.2)*·*10^−3^ s^−1^ at 25°C in 0.05 M phosphate buffer,  pH 7.0, was calculated using kinetic modeling based on the experimental data. Both reactions are reversible. Spectrophotometry and mass-spectrometry methods have firmly shown that the penicillamine ligand is exchanged for GS^−^ during decomposition of 1.5*·*10^−4^ M (**I**) in the presence of 10^−3^ M GSH, with 76% yield in 24 h. As has been established, such behaviour is caused by the resistance of (**II**) to decomposition due to the higher affinity of iron to GSH in the complex. The discovered reaction may impede S-glutathionylation of the essential enzyme systems in the presence of (**I**) and is important for metabolism of NIC, connected with its antitumor activity.

## 1. Introduction

The intensive recent research in fundamental nitrogen monoxide (NO) chemistry as one of the necessary and universal regulators of cellular metabolism functions [1, 2] includes investigation of the properties of synthetic nonheme nitrosyl iron complexes (NICs) biomimetics of cellular NO-intermediates [3–8]. NICs with functional sulfur-containing ligands represent a class of efficient NO-donating compounds [9] that can be used as a basis for new-generation medicinal products: efficient antitumour agents [10–12], original vasodilating medicinal products to treat acute coronary syndrome [13–16], and so forth. In the case of NO therapy it is especially important to study mechanisms of action of synthetic NICs and their biotransformation* in vivo* as potential NO-donating drugs (antitumor oncolytics, antihypertension, and antiaggregation action) and on functions of physiologically significant heme proteins. Intense studies of new exogenic donors of nitrogen monoxide (NO), carried out in recent years, have been directed to create new ways of producing NO. Clinical use of formulations that produce NO under physiological conditions has serious disadvantages related to their nitrate tolerance. Models of active centres of nonheme iron-sulfur proteins, NICs with functional sulphur-containing ligands synthesized by the Chemical Physics Institute of Russian Academy of Science [17, 18], spontaneously evolve NO in aqueous medium as a result of decomposition and appear to be prospective promedicines of a new generation [19, 20]. These are substances with dual drug-induced effect caused by biologically active thiols on the one hand and nitrogen monoxide on the other. They showed antitumour and cardiologic activity in preclinical testing.

We found that complex (**I**), the synthesis and composition of which are described [14, 21], evolves NO and penicillamine thiolyl upon decomposition [21].

The goal of this paper was the kinetic modeling of the decomposition of (**I**) to determine the elimination rate constant of penicillamine. The study also aimed to investigate the exchange reaction of the thiol ligands of (**I**) (as an efficient exogenic donor of NO [14]) for glutathione, as this is an important process for a possible exchange reaction of the NIC thiol ligand with biologically significant thiols* in vivo*. We have chosen reduced glutathione (GSH) to study ligand exchange reactions with (**I**). GSH is a water-soluble tripeptide consisting of amino acids: glutamic acid, cysteine, and glycine. GSH is the most commonly encountered nonprotein thiol in animal, and concentration in human tissues varies from 0.1 to 10 mM. The highest concentration is found in the liver, spleen, kidneys, crystalline lens, erythrocytes, and leucocytes. The functions of GSH are vital and versatile. Its cysteine thiol acts as a nucleophile in reactions with endogenous and exogenous compounds. Its main functions are (1) antioxidant, (2) cofactor of numerous cytoplasmic enzymes, and (3) thiolating agent at significant posttranslation modification of a number of cellular 3 proteins. The correlation between metabolism of GSH and such diseases such as cancer, neurodegenerative diseases, cystic fibrosis, HIV, and aging [22] has been established. Moreover GSH may promote S-glutathionylation of essential enzymes, receptors, structural proteins, transcription factors, and transport proteins [23].

## 2. Experimental

### 2.1. Materials

We used bovine Hb, Tris (Serva, Germany), acetonitrile LC-MS grade (Panreac, Spain), reduced L-glutathione, KI (ALDRICH, USA), Na_2_HPO_4_·6H_2_O and NaH_2_PO_4_·H_2_O (MP Biomedicals, Germany), FeSO_4_·7 H_2_O, D-penicillamine (SIGMA, USA), Sephadex G-25 (Pharmacia, Sweden), and sodium dithionite (Merck, Germany). Water was purified by distillation in a Bi/Duplex distiller (Germany).

**Figure 1:**
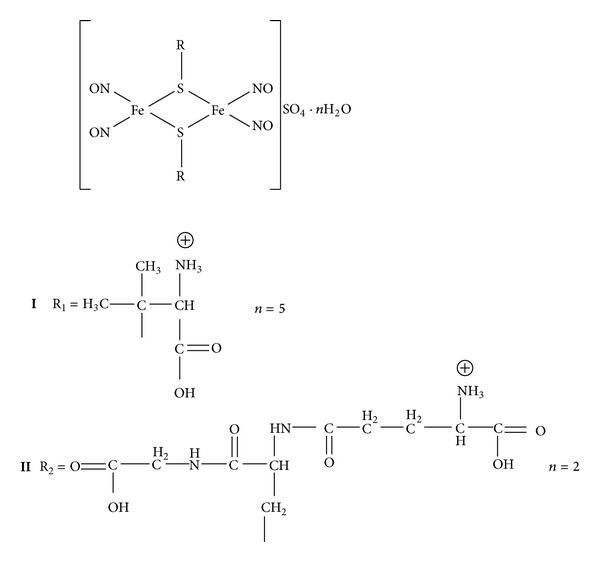
Chemical structures of the tetranitrosyl iron complexes (**I**) and (**II**) [14].

The synthesis, structure (CCDC 680286), and physicochemical data of (**I**) (Figure 1) have been described elsewhere [14, 21] and (**II**) was synthesized by a similar method [14]. Complex (**I**) has been obtained by reaction of ferrous sulphate(II) with an aqueous solution of D-penicillamine in the molar ratio 1 : 3. The reaction was performed using a standard vacuum line and Schlenk technology under argon. Previously, oxygen has been removed from the water by triple freezing and vacuum pumping. To a dry mixture, containing 0.42 g (1.5 mmol) of FeSO_4_·7 H_2_O and 0.68 g (4.5 mmol) D-penicillamine were added 10 mL water, prepared as described above, and nitric oxide was passed through the resulting deep purple solution at room temperature. Fine red needles appeared on the walls of the reaction vessel after 10–12 min and gradually filled the entire volume of the solution. The mixture was kept for 3 days at 6–8°C, filtered, and dried in vacuum under argon. Yield is 198 mg (20%). Complex (**II**) was prepared by the same synthetic route. Elemental analysis, Mössbauer, and EPR spectroscopy confirmed that the structure is similar to that of complex (**I**), but instead of the penicillamine thiol ligands two molecules GSH are present [21].

*Elemental analysis* of (**I**) and (**II**)* polycrystals* was conducted at the Multiaccess Analytic Centre of IPCP RAS.

*IR-spectrum *(cm^−1^) of (**I**) and (**II**) was recorded on a PerkinElmer Spectrum 100X at room temperature. For (**I**)  found: Fe, 15.60; C, 16.72; H, 4.50; N, 11.75; O, 38.02; S, 13.40%. Fe_2_C_10_H_32_N_6_O_17_S_3_ required: Fe, 15.64; C, 16.76; H, 4.47; N, 11.73; O, 37.99; S, 13.41%; IR: *ν*/cm^−1^ = 1771 (s, NO); 1723 (s, NO). For (**II**) found: Fe, 11.40; C, 24.52; H, 3.91; N, 14.28; O, 36.02; S, 9.79%. Fe_2_C_20_H_38_N_10_O_22_S_3_ required: Fe, 11.45; C, 24.54; H, 3.89; N, 14.31; O, 35.99; S, 9.82%. IR: *ν*/cm^−1^ = 1771 (s, NO); 1728 (s, NO).

### 2.2. Operation Technique in Inert Gas Atmosphere [24]

All procedures were carried out under nitrogen (high purity grade), which was additionally purified by passing through a column with a chromium-nickel catalyst. Nitrogen was purged to the magnetically stirred working solutions (buffer, water) solution for 30 min. Hereinafter these solutions are named anaerobic. All the vessels used and quartz cells were sealed with Rubber Septa seals (Sigma, USA), which allowed one to introduce gases and other necessary components through a needle. A solution was transferred from one vessel to another using syringes with soldered needles or under excess nitrogen pressure using two needles connected with Teflon capillaries. Excess pressure was discharged through an additional needle capped with a Teflon capillary immersed into water. Cells and vessels with a volume of 4 and 5 mL, respectively, containing weighed samples of the NICs or other reagents were purged with nitrogen through needles for 30 min.

### 2.3. Electrochemical Determination of the Concentration of NO Evolved by **(I)** in Solutions under Examination

Amperometric sensor electrode amiNO-700 of in NO Nitric Oxide Measuring System (Innovative Instruments, Inc., USA) was used to measure the concentration of NO generated by (**I**). NO concentration in water solution was recorded for ca 500 s (using 0.2 s increments) with NO-donor concentration in the solution equaling 0.4 · 10^−5^ M. The experiments were conducted under nitrogen atmosphere. A standard water solution of NaNO_2_ (0.01 M), supplemented with an aqueous solution of 20 mg of KI and 2 mL of 1 M H_2_SO_4_ (c.p.) in 18 mL of water as recommended by the manufacturer [25], was used to calibrate the electrochemical sensor. Sensor calibration and experiments were carried out at 25°C with intensive stirring. Phosphate buffer 0.05 M pH 7.0 was used. Oxygen was removed from the buffer for experiments in anaerobic conditions by triple freezing and degassing in vacuum using standard Schlenk line technique, after which the buffer was stored up to 24 h in glass-stoppered flasks. To the sample of (**I**) in nitrogen-filled vessel 0.05 M anaerobic phosphate buffer pH 7.0 in order to obtain (**I**) 6 · 10^−4^ M solution was added and stirred for 10 to 15 min until complete dissolution of (**I**) shot. At the same time inert gas was transmitted for 30–40 minutes through the measuring electrochemical cell equipped with Rubber Septa (Sigma, USA) seals and connected to a thermostat, containing 49.5 mL of the prepared phosphate buffer with immersed thermal sensor and electrode. After this 0.5 mL of solution was removed from the vessel containing (**I**) solution and fed into the measuring cell though a rubber seal. Recording the formation of NO in the system was started at the same time.

### 2.4. Preparation of an Hb Solution

A homogeneous preparation of bovine Hb was obtained from bovine hemoglobin (MP Biomedicals, Germany), which was a mixture of methemoglobin (metHb) and oxygenated hemoglobin (HbO_2_). A 0.05 Mphosphate buffer (pH 7.0) was used at all stages of Hb preparation and in all experiments with Hb. To convert a mixture of metHb with HbO_2_ to Hb, a column 2 × 15 cm packed with Sephadex G-25 was prepared and transformed into the anaerobic state. For this purpose, 50 mL (volume of the column) of the anaerobic buffer and then 40 mL of the buffer containing sodium dithionite (5 mL, 100 mg mL^−1^) were passed through the column. The column was left to stand for 3 min and then dithionite was washed out with 50 mL the anaerobic buffer until a negative reaction for dithionite (with methyl viologen) was achieved. Commercial hemoglobin (0.5 g) was dissolved with stirring in the buffer (5 mL), nitrogen was purged for 30 min with stirring, and a solution of dithionite (2 mL, 100 mg mL^−1^) was added. The absorption spectrum of an aliquot of the solution showed that the whole mixture of metHb with HbO_2_ had been transformed into Hb. Then excess dithionite and products of its decomposition were removed on a Sephadex G-25 column. A solution of Hb (5 mL) with a concentration of ~6 · 10^−4^ M was eluted. The solution of Hb was stored in the frozen state as aliquots in liquid nitrogen. Prior to use the Hb solution was thawn out in 5 mL flasks in a nitrogen flow. The indicator of purity and homogeneity of Hb was the ratio of molar absorption coefficients of all absorption maxima coinciding with published data.

### 2.5. Decomposition of Complex **(I)** or **(II)** at pH 7.0

The experiments were carried out using the same original 6 · 10^−4^ M solution of NIC. To the sample of NIC in a nitrogen-filled vessel 0.05 M anaerobic Tris-HCl buffer pH 7.0 in order to obtain a NIC 6 · 10^−4^ M solution was added, which was dissolved for 15 min and then frozen in liquid nitrogen in the shape of balls. For the purpose of experiments NIC was thawed under nitrogen flow for about 20 minutes until complete melting of the balls, and then solution aliquots of 0.75 mL were taken and inserted in a 4 mL anaerobic test cuvette (1 cm of optical path), containing 2.25 mL of 0.05 M anaerobic buffer pH 7.0 to achieve NIC final concentration of 1.5 · 10^−4^ M. The reference cuvette contained 3 mL of buffer. The absorption spectra were recorded between 250–500 nm and 300–650 nm range at appropriate time intervals at 25°C.

### 2.6. Kinetics of **(I)** Interaction with GSH

The experiments were carried out under nitrogen atmosphere.** A **6 · 10^−4^ M (**I**) solution, prepared as described above was used for experiments and 10^−2^ M GSH solution in 0.1 M Tris-HCl buffer pH 7.0. A 1.95 mL of anaerobic buffer and 0.75 mL of 6 · 10^−4^ M (**I**) solution were inserted in a 4 mL anaerobic test cuvette. The reaction was initiated by adding 0.3 mL of 10^−2^ M GSH solution. The final concentration of (**I**) in test cuvette was 1.5 · 10^−4^ M. The reference cuvette contained anaerobic buffer pH 7.0 and (**I**) of the same concentration as in the test cuvette. The difference absorption spectra were registered at appropriate intervals, as indicated in the figures.

### 2.7. Kinetics of NIC Interaction (**(I)** or **(II)**) with Hb

We used 6 · 10^−4^ M solutions of either (**I**) or (**II**) in 0.05 M anaerobic Tris-HCl-buffer pH 7.0 after defrosting under nitrogen flow, prepared as described above. A 0.75 mL of NIC solution under nitrogen transferred to an anaerobic test cuvette and a 4 mL comparison cuvette, containing such quantity of 0.05 M anaerobic buffer pH 7.0, so that the resulting volume of reaction solution after introduction of approximately 0.11 mL of Hb 5.4 · 10^−4^ M solution into test cuvette would be 3.0 mL. The reaction was initiated by adding Hb solution to the test cuvette to reach a 2 · 10^−5^ M concentration. Final concentration of NIC solution in the test cuvette and reference cuvette was 1.5 · 10^−4^ M. Further the difference absorption spectra were registered at appropriate intervals, as indicated in the figures. Similarly the interaction of Hb with NIC 1.5 · 10^−4^ M in the presence of GSH 10^−3^ M in anaerobic Tris-HCl buffer pH 7.0 was studied. The buffer solution was inserted into anaerobic cuvettes (1.84 mL and 1.95 mL in the test and reference cuvette, resp.), 0.75 mL NIC 6 · 10^−4^ M, and 0.3 mL of a 10^−2^ M GSH solution in 0.1 M Tris-HCl buffer pH 7.0. The reaction was initiated by adding the Hb solution in the test cuvette up to a 2 · 10^−5^ M. Then the difference absorption spectra were registered at appropriate intervals, as indicated in the figures.

### 2.8. Absorption Spectra

Absorption spectra were recorded at 25°C using a Specord M-40 spectrophotometer equipped with an interface for computer-aided registration of spectra and thermostatic cuvette holder.

### 2.9. Amount of Hb and HbNO

Amount of Hb and HbNO was evaluated spectrophotometrically. For this purpose absorption spectra were factored by components as described in the paper [17]. To determine the HbNO concentration, the absorption spectrum of the reaction system containing Hb and HbNO was deconvoluted to the components (spectra of Hb and HbNO) by computer processing using the MathCad program. The solution should satisfy the criterion of the minimum of the sum of squared deviations of the experimental spectrum of the mixture from the calculated one:

(1) $$\begin{array}{c} \sigma \left(\alpha ,\beta \right)=\sum_{i}{\left[D_{i}-F\left(D_{\text{Hb}},D_{\text{HbNO}},\alpha ,\beta \right)\right]}^{2}, \end{array}$$

where *σ*(*α*, *β*) is the root mean square deviation; *D*_*i*_  are the experimental data (absorbance) at a certain time moment; *F* is the desired function of the *D*_Hb_, *D*_HbNO_, *α*, and *β* values; *D*_Hb_ and *D*_HbNO_ are the initial absorbances of Hb and HbNO, respectively; *α* and *β* are the fractions of HbNO and Hb, respectively. The calculation was performed in a wavelength region of 450–650 nm by 200 experimental points. In the whole series of experiments, the *σ*(*α*, *β*) value ranges from 2 · 10^−5^ to 5 · 10^−6^, indicating high quality of simulation of the absorption spectrum of the reaction mixture at each moment and high accuracy of determination of the Hb and HbNO concentrations.

### 2.10. Mass-Spectrometric Analysis

Mass-spectrometric analysis was carried out using a 2020 Shimadzu LC-MS instrument that includes a liquid chromatograph LC-20 Prominence with matrix photo detector SPD-M20A (200–800 nm) and mass-selective quadrupole detector (*m*/*z* scanned mass range: 50–2000; ionization modes: DUIS/ESI/APCI). Analysis conditions were as follows: ionization method-electrospray ionization, ESI-MS, sample input method-direct input, solvent-acetonitrile, incubation (25°C), exposure mode-positive mode, or negative mode. Analysis samples were prepared under nitrogen atmosphere in 0.05 M Tris-HCl-buffer pH 7.0. 2 mL vessels with a PTFE/Silicone/PTFE seal allow samples to be inserted with a syringe, blown with nitrogen for about 10 minutes before the sample was inserted.

### 2.11. ^57^Fe Mössbauer Absorption Spectra of **(I)** and **(II)** Were Recorded on WissEl Operating in Constant Acceleration Mode

^57^Co in Rh matrix was used as the source. Spectra at low temperatures were measured using continuous flow helium cryostat CF-506 (Oxford Instruments) with controllable temperature. Mössbauer spectra were processed by the least square method assuming the Lorentzian form of the individual spectral components.^ 57^Fe Mössbauer spectra of polycrystals of (**I**) and (**II**) are a single doublets; with the parameters (quadrupole splitting Δ*E*_*Q*_ = 0.913(4) mm/s, isomer shift *δ*_Fe_ = 0.085(2) mm/s, width of absorption lines 0.272(1) mm/s for (**I**) and Δ*E*_*Q*_ = 1.009(3) mm/s, *δ*_Fe_ = 0.076(2) mm/s, width of absorption lines 0.250(1) mm/s for (**II**) at temperature 85 K). They are similar to those for other complexes of “*μ*-S” structural type. This indicates the structural equivalence of two iron atoms in (**I**) and (**II**).

### 2.12. The Proposed Reaction Scheme Describing Decomposition of **(I)** Was Considered for Kinetic Modelling

The values of the rate constants were determined using the least squares method on the basis of a numerical solution of the respective system of differential equations. The concentrations of NO and (**I**), determined by a sensor electrode or absorption spectra, were used as experimental data.

## 3. Results and Discussion

### 3.1. Decomposition of **(I)**

Complex (**I**) (Figure 1) spontaneously evolves NO as a result of decomposition. This paper covers decomposition of (**I**) using two methods: (1) by direct determination of quantity of the released NO by means of sensor electrode and (2) spectrophotometrically by observing the absorption spectrum variation of (**I**).

#### 3.1.1. Decomposition of **(I)** in 0.05 M Phosphate Buffer pH 7.0. Measurement of NO Using a Sensor Electrode

**Figure 2:**
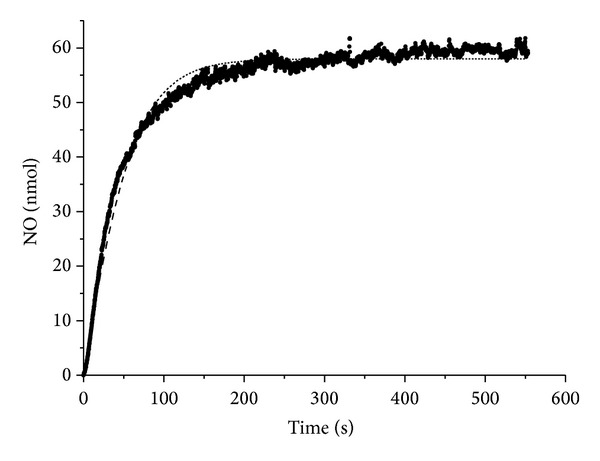
Kinetic curve of the accumulation of NO in solution (the measurements were carried out by a sensor electrode) in the course of the decomposition of (**I**) 4 · 10^−6^ M in 0.05 phosphate buffer pH 7.0 under anaerobic conditions at 25°C. The dashed line represents the approximation by *dx*/*dt* = *k*_1_ · [(**I**)]_0_ − *k*_1_ · *x* − *k*_*n*_ · *x* ^2^. (**I**) is the complex [Fe_2_(SC_5_H_11_NO_2_)_2_(NO)_4_]SO_4_ · 5H_2_O.

Sensor electrode permits to measure total amount of NO in system, in solution, and adsorbed on the electrode: *x*(*t*) = [NO] + NO_*s*_/*V*. *V* is the volume of reaction mixture in electrochemical cell equaling 50 mL. [NO] is the NO concentration in the solution and NO_s_ is the number of NO gram-molecules on the electrode.  Figure 2 represents the kinetic curve of space missing NO accumulation in a cell as recorded by a sensor electrode. Initial concentration of (**I**) is equal to 4 · 10^−6^ M. It is seen that the curve rapidly reaches plateau, which is far less than  [(**I**)]_0_. It shows that evolution of NO is reversible and the reaction system is in a state of equilibrium:

(2) $$\begin{array}{c} \left(I\right)\overset{\left(k_{1},k_{-1}\right)}{⇌}{\text{P}}_{1}+\text{NO} \end{array}$$

NO binding to the electrode is the equilibrium process:

(3) $$\begin{array}{c} \text{NO}\overset{\left(k_{s},k_{-s}\right)}{⇌}{\text{NO}}_{s} \end{array}$$

Equation of NO accumulation is as follows:

(4) $$\begin{array}{c} \frac{dx}{dt}=k_{1}\cdot {\left[\left(I\right)\right]}_{0}-k_{1}\cdot x-k_{n}\cdot x^{2}. \end{array}$$

The required parameters are *k*_1_ and *k*_*n*_ = *k*_−1_/(1 + *K*_*s*_), where *k*_*s*_ is the equilibrium constant of binding NO to the electrode. Parameter *k*_*n*_ describes the reversibility of (**I**) decomposition in the presence of NO binding to the electrode. As the NO amount, adsorbed on the electrode is much less than the amount of NO in solution (it is the property of this electrode), *K*_*s*_ ≪ 1, *k*_*n*_ ≈ *k*_−1_. As a result of approximation of the experimental curve with (4) the following values of required rate constants have been obtained: *k*_1_ = (4.6 ± 0.1) · 10^−3^s^−1^; *k*_*n*_ = (9.7 ± 0.2) · 10^−3^ M^−1^ · s^−1^ (Figure 2).

#### 3.1.2. Decomposition of **(I)** in 0.05 M Phosphate Buffer pH 7.0. Measurement of **(I)** Concentration Using Spectrophotometry

**Figure 3:**
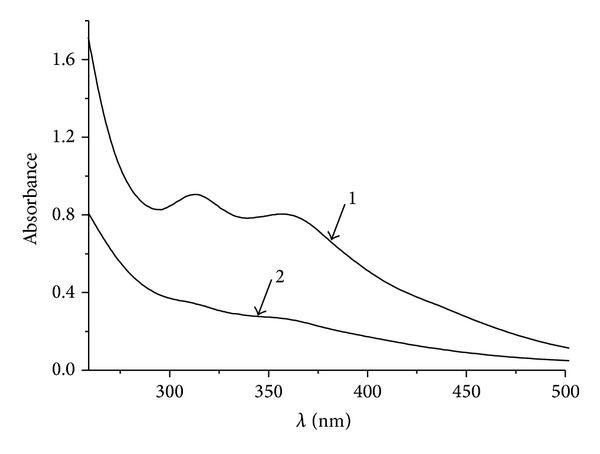
Spectra of 10^−4 ^M (**I**) in 0.05 M phosphate buffer, pH 7.0 makeup (1) and after 18 hours at 25°C (2). Extinction at *ƛ* = 312 nm is equal to 9.1 · 10^3^ M^−1^ cm^−1^ and 2.6 · 10^3^  M^−1^ cm^−1^ at 450 nm. (**I**) is the complex [Fe_2_(SC_5_H_11_NO_2_)_2_(NO)_4_]SO_4_ · 5H_2_O.

**Figure 4:**
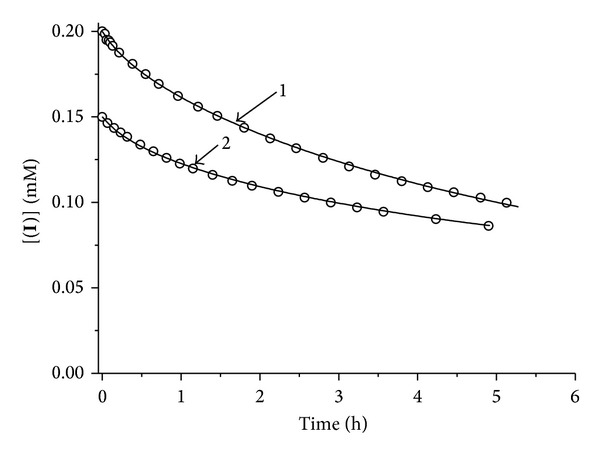
Kinetics of (**I**) decomposition in 0.05 M phosphate buffer, pH 7.0 at 25°C. Starting (**I**) concentrations are equal to 1.5 · 10^−4^(1) and 2 · 10^−4^(2) M. Circles are the experimental data. Solid lines are the simulated curves, corresponding to the experimental points. Simulation was made by means of a system of differential equations (6). (**I**) is the complex [Fe_2_(SC_5_H_11_NO_2_)_2_(NO)_4_]SO_4_ · 5H_2_O.

The change of absorption spectra of (**I**) was monitored during this experiment. Absorption spectra within 450 to 650 nm range were recorded at 10–15 min intervals and, keeping in mind the molar absorption extinction of (**I**) in the maximum absorption point in this range—450 nm (Figure 3), we built kinetic relations of absorbance change (decrease) substrate concentration (Figure 4). It has been established that in anaerobic conditions the rate of decomposition increases subject to increase in the concentration of (**I**) (Figure 4). However, in case of (**I**) the change in absorption spectra takes places not only due to NO-group evolution, but by elimination of penicillamine ligand (L). Assuming that ligand is eliminated both from (**I**) and P_1_ product, resulting after NO evolution from (**I**), the following reaction mechanism can be suggested:

(5) $$\begin{array}{c} \left(I\right)\overset{\left(k_{1},k_{-1}\right)}{⇌}{\text{P}}_{1}+\text{NO},\;\;{\text{P}}_{1}\overset{\left(k_{2},k_{-2}\right)}{⇌}{\text{P}}_{2}+\text{L},\\ \left(I\right)\overset{\left(k_{3}\right)}{⇌}{\text{P}}_{3}+\text{L} \end{array}$$

The results obtained with sensor electrode suggest that *k*_1_ = (4.6 ± 0.1) · 10^−3^ s^−1^; *k*_*n*_ = (9.7 ± 0.2) · 10^3^ M^−1^ · s^−1^. Required parameters are *k*_2_, *k*_−2_, *k*_3_. (**I**) and P_1_ are spectrophotometrically indistinguishable, so experimentally measured value *x*(*t*) = [(**I**)] + [P_1_].

The system of equations, corresponding to scheme of reactions:

(6) $$\begin{array}{c} \frac{d\left[I\right]}{dt}=-k_{1}\left[I\right]+k_{-1}\left[{\text{P}}_{\text{1}}\right]\left[\text{NO}\right]-k_{3}\left[I\right]\\ \frac{d\left[\text{NO}\right]}{dt}=k_{1}\left[I\right]-k_{-1}\left[{\text{P}}_{\text{1}}\right]\left[\text{NO}\right]\\ \frac{d\left[{\text{P}}_{\text{1}}\right]}{dt}=k_{1}\left[I\right]-k_{-1}\left[{\text{P}}_{\text{1}}\right]\left[\text{NO}\right]-k_{2}\left[{\text{P}}_{\text{1}}\right]+k_{-2}\left[{\text{P}}_{\text{2}}\right]\left[\text{L}\right]\\ \frac{d\left[{\text{P}}_{\text{2}}\right]}{dt}=k_{2}\left[{\text{P}}_{\text{1}}\right]-k_{-2}\left[{\text{P}}_{\text{2}}\right]\left[\text{L}\right]\\ \frac{d\left[\text{L}\right]}{dt}=k_{2}\left[{\text{P}}_{\text{1}}\right]-k_{-2}\left[{\text{P}}_{\text{2}}\right]\left[\text{L}\right]+k_{3}\left[I\right]. \end{array}$$

By solution of system of equations using the kinetic modelling method, the values of required rate constants (Table 1) have been found that satisfactorily describe the curves of Figure 4 (approximation is shown in solid lines). Based on Table 1 *k*_2_ (reaction rate of penicillamine ligand eliminated from (**I**)) is determined with good accuracy. As experimental data error is ±10%, the value of *k*_2_ = (1.8 ± 0.2) · 10^−3^ s^−1^.

### 3.2. Ligand Exchange in **(I)** for GSH Based on Spectrophotometric Data

**Figure 5:**
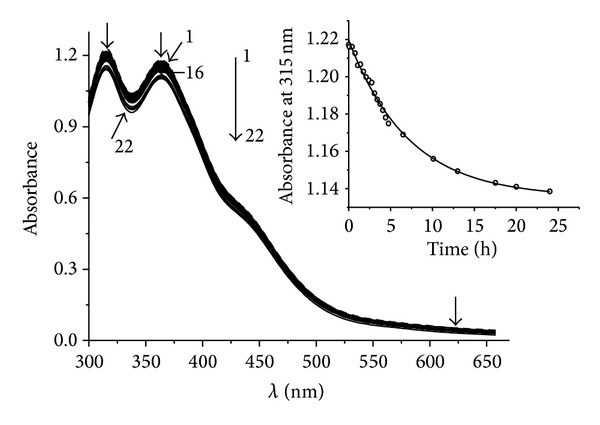
Kinetics of change of absorption spectrum of (**II**) (1.5 · 10^−4^ M): spectra were registered at 30 s (1), 5 (2) min after start of reaction. Spectra 3–16 were registered further with interval 20 min. Spectra 17–22 were registered at 6.5 (17), 10.1 (18), 13 (19), 17.5 (20), 20 (21), and 24 (22) h after start of reaction. Conditions of reaction: 25°C, solvent is 0.05 M Tris-HCl buffer, and pH 7.0. Spectra 1–22 have 2 maxima: *λ*_1_ = 315 nm and *λ*_2_ = 365 nm; *ε*_315 nm_ is equal to 8.2 · 10^3^ M^−1^·cm^−1^. The inset shows kinetics of (**II**) (1.5 · 10^−4^ M) decomposition in 0.05 M Tris-HCl-buffer pH 7.0 at 25°C (for the experimental data shown on this figure). Circles are experimental data. Approximation (theoretical curve) was made by means of *y*(*t*) = *y*_*o*_ + *A* · *e* ^−*kt*^. (**II**) is the complex [Fe_2_(SC_10_H_17_N_3_O_6_)_2_(NO)_4_]SO_4_ · 2H_2_O.

**Figure 6:**
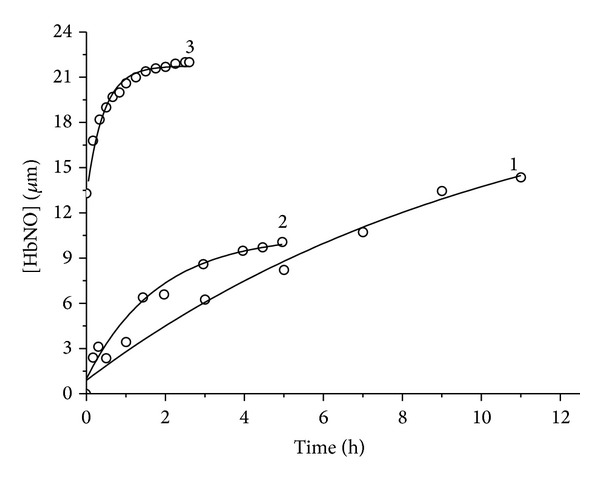
(1) Kinetics of HbNO formation at interaction of (**II**) with Hb on the base of the experimental data shown in (a). (2) Kinetics of HbNO formation at interaction of (**I**) with GSH in Hb presence. Hb on the base of the experimental data shown in (b). (3) Kinetics of HbNO formation in the interaction of (**I**) with Hb, the experimental data shown in (c). Circles are the experimental data. Solid line is the approximation by means of *y*(*t*) = *y*_*o*_ + *A* · (1 − *e* ^−*kt*^). (Figures a, b, and c are in Supplementary Materials available online at http://dx.doi.org/10.1155/2014/641407). (**II**) is the complex [Fe_2_(SC_10_H_17_N_3_O_6_)_2_(NO)_4_]SO_4_ · 2H_2_O.

In this paper we investigated the interaction of (**I**) with GSH. In addition to the importance of GSH, this choice is also explained by the fact that we intended to obtain an NIC product of the same structure as (**I**) (Figure 1) but with GSH as the thiolic ligand,* bis*-(glutathione-2-thiolate) tetranitrosyl diiron (**II**). According to our data, (**II**) proved to be extremely resistant to decomposition (Figure 5). We used 0.05 M Tris-HCl buffer as a solvent due to the simultaneous mass-spectrometric analysis of samples being conducted. In the phosphate buffer, where NIC was dissolved earlier [17], the phosphate spectrum superimposed the test sample's spectrum in the profile of multiple peaks. All investigations were conducted under nitrogen atmosphere, as NO promptly interacts with O_2_, producing nitrogen oxides with rate constant 2 · 10^6^(M^−1^)^2^ · s^−1^ at 25°C [26]. Figure 5 shows data concerning the change in absorption spectrum of (**II**) in solution, whereas Figure 6 (curve 1) shows the kinetics of NO evolution by formation of HbNO.

**Figure 7:**
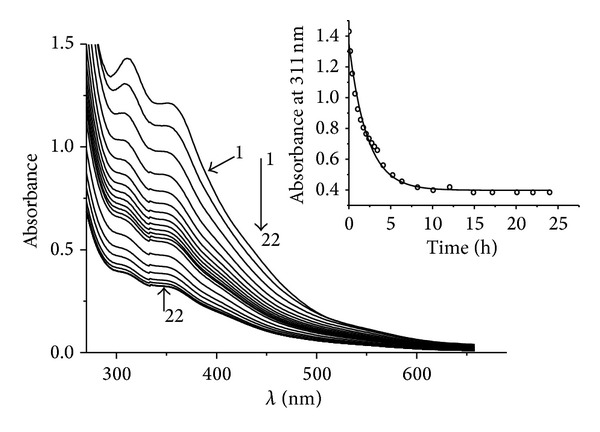
Kinetics of change of absorption spectrum of (**I**) (1.5 · 10^−4^ M): spectra were registered at 30 s (1), 9 min (2) after start of reaction. Spectra 3–12 were registered further with interval of 20 min. Spectra 13–22 were registered at 4.1 (13), 5.2 (14), 6.3 (15), 8.2 (16), 10 (17), 14.9 (18), 17.1 (19), 20.1 (20), 22 (21), and 24 (22) h after start of reaction. Conditions of reaction: 25°C solvent is 0.05 M Tris-HCl buffer, pH 7.0. Spectra 1–22 have 2 maxima: *λ*_1_ = 311 hm *и* *λ*_2_ = 353 hm. The inset shows kinetics of the decomposition of (**I**) (1.5 · 10^−4^ M) in 0.05 M Tris-HCl-buffer pH 7.0 at 25°C (for the experimental data shown on this figure). Circles are the experimental data.

**Figure 8:**
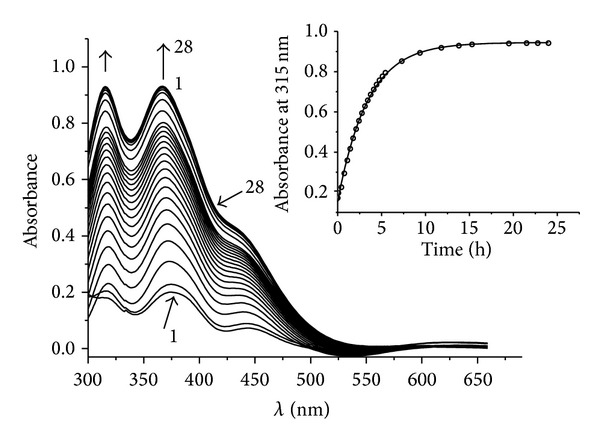
Kinetics of absorption changes in the interaction of (**I**)  (1.5 · 10^−4^ M) with GSH (10^−3 ^M): spectra were registered at 45 s (1), 5 min (2), and 20 min (3) after start of reaction. Spectra 4–18 were registered further with interval 20 min. Spectra 19–28 were registered at 7.3 (19), 9.4 (20), 11.8 (21), 13.8 (22), 16.3 (23), 19.5 (24), 21.5 (25), 22.8 (26), 23 (27), and 24 (28) h after start of reaction. Conditions of reaction: 25°C, solvent is 0.05 M Tris-HCl buffer, pH 7.0. Spectra 1–28 have 2 maxima: *λ*_1_ = 315 nm and *λ*_2_ = 367 nm. The inset shows kinetics of (**II**)  accumulation at interaction of (**I**)  with GSH on the base of the experimental data shown on this figure. Circles are the experimental data. Approximation (theoretical curve) was made by means of *y*(*t*) = *y*_o_ + *A* · *e* ^−*kt*^. (**I**)  is the complex [Fe_2_(SC_5_H_11_NO_2_)_2_(NO)_4_]SO_4_ · 5H_2_O, and (**II**) is the complex [Fe_2_(SC_10_H_17_N_3_O_6_)_2_(NO)_4_]SO_4_ · 2H_2_O.

For comparison, the same investigation was carried out with (**I**) (Figure 7, Figure 6 curve 3). Hb demonstrates a specific absorption spectrum that alters as NO is attached. Therefore, as described in papers [14, 17] for this class of NO donors, evolution of NO can be traced by formation of HbNO. Since all NICs absorb in the visible spectrum, the experiment recorded difference absorption spectra of the buffer and test system with Hb containing NIC in equal concentrations. The composition of reaction mixtures is described in Experimental. By recording HbNO accumulation and fractioning of absorption spectra into components, we measured the kinetics of HbNO formation (Figure 6). Using the following equation: *y*(*t*) = *y*_*o*_ + *A*(1 − *e* ^−*kt*^), we obtained effective first-degree rate constants (*k*) for these reactions (given in Table 2). It has been established that NO is evolved from (**II**) almost 25 times more slowly than from (**I**) (Table 2, Figure 6), while absorption spectrum of (**II**) decreases 3.4 times more slowly as compared to that of (**I**) (Figures 5 and 7). In the reaction scheme containing (**I**) and GSH (Figure 8) the absorption spectrum, the parameters of which match the absorption spectra of (**II**) (Figure 5), grew. Maximum absorption increase took place within 24 hours. At the same time the concentration of the resulting (**II**) (taking into account that *ε* at 315 nm is equal to 8.18 · 10^3^ M^−1^ cm^−1^, Figure 5) was 1.15 · 10^−4^ M, while concentration of the original (**I**) is 1.5 · 10^−4^ M; that is, the output is 76%. Output cannot reach 100% because the (**II**) decay takes place in parallel and output of (**II**) should be determined by the ratio of the equilibrium constants of (**I**) and (**II**). When Hb was present in the (**I**)-GSH system, HbNO accumulated with *κ* equal 1.5 · 10^−4^ s^−1^ (Figures 6, curve 2); that is, NO output rate was somewhere between (**I**)-Hb (Figure 6 curve 3) and (**II**)-Hb (Figures 6, curve 1) system as NO evolved both from (**I**) and (**II**).

As seen in Figure 6, curve 3, there are some traces of HbNO (the reaction was initiated by adding Hb) at time zero, with initial concentration for (**I**) being higher. This can be explained by the fact that dissolution of NIC (see Experimental) took a certain time, ~15 minutes. Partial decomposition with release of NO took place during this period of time, and then newly formed HbNO was detected at the first recording of the absorption spectrum. Original NIC solution (6 · 10^−4^ M) was frozen to ensure an NIC solution of the same concentration was used.

### 3.3. Mass-Spectrometry Analysis

Mass-spectral analysis of the reaction mixture of (**I**) with GSH (Figure 8, Table 3) was performed. In the course of analyzing the products of the interaction of (**I**) with GSH (Figure 8) after a 24 h incubation at 25°C, which corresponded to maximum output of the product of the interaction of these compounds (Figure 8), the (**II**) cation was detected. Moreover, the spectrum shows a certain amount of the products of decomposition of (**II**) and also the oxidized form of GSH, GS-SG. Thus, the results of mass-spectral analysis of reaction mixture of (**I**) with GSH qualitatively correspond to the data obtained in the spectrophotometric study.

## 4. Conclusions

This paper shows for the first time that NIC with a thiol ligands of penicillamine [Fe_2_(SC_5_H_11_NO_2_)_2_(NO)_4_]SO_4_ · 5H_2_O (**I**) reversibly releases both NO and thiol ligand in aqueous medium. Rate constants of these first-degree reactions were determined. Decomposition equilibrium of (**I**) apparently shifts as NO is consumed in reactions that are required as universal regulators of cellular metabolism functions [1, 2]. Convincing evidence shows that penicillamine ligands of NIC (**I**) in solution in the presence of GSH replaces the original thiolic ligands with GS-, thus forming new NIC, (**II**), which is quite decomposition-resistant and shown here. We assume that this may influence the important role of (**I**) in biotransformations, connected with antitumour activity. The strength of the thiol-Fe bond in (**II**) is impressive. It can probably be explained by the fact that GSH is a tripeptide and is bonded with the S-group of cysteine, which is located between glutamic acid and glycine. These two amino acids likely “shield” the Fe-S bond in (**II**) from attacks by thiol and water.

## Supplementary Material

Kinetics of change of difference spectra at the interaction of ( I ) and ( II ) with Hb.
( I ) complex [Fe_2_(SC_5_H_11_N*О*_2_)_2_(NO)_4_]SO_4_ *∙*5H_2_O.
( II ) complex [Fe_2_(SC_10_H_17_N_3_O_6_)_2_(NO)_4_]SO_4_•2H_2_O).

## Figures and Tables

**Figure 9:**
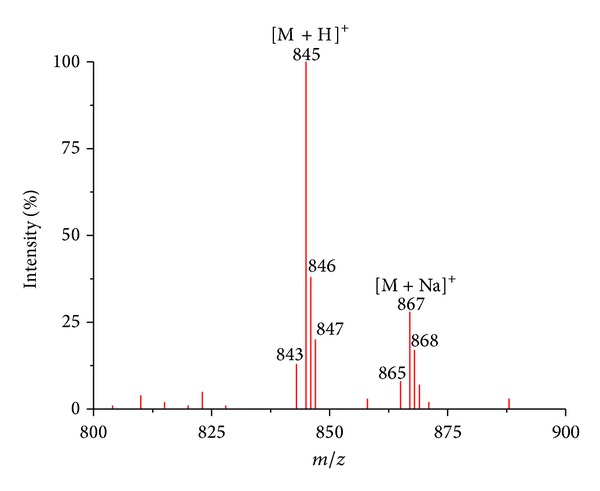
Mass spectrum (**I**)  + GSH (as in experiment, shown in Figure 8) after 24 h from the start of the reaction: (ESI, +4.5 kV). (**I**)  is the complex [Fe_2_(SC_5_H_11_NO_2_)_2_(NO)_4_]SO_4_ · 5H_2_O. M is the cation of (**II**).

**Table 1:** Results of kinetic modelling calculations (denote the rate constants of the reactions that are given in the text).

[(**I**)]_0_ · 10^4^, M	*k*_2_ · 10^3^, s^−1^	*k*_−2_, M^−1^·s^−1^	*k*_3_ · 10^5^, s^−1^
1	1.9	0.1	0.8
1.5	1.7	0.2	0.9
2.0	1.8	0.1	1.8

**Table 2:** Results of kinetic experiments (average of three).

Figure number	NIC	Process	*k*, s^−1^
Figure 5	(**II**)	Decomposition	(3.8 ± 0.4) · 10^−5^
Figure 6	(**II**)	Interaction with Hb	(2.6 ± 0.3) · 10^−5^
Figure 6	(**I**)	Interaction with Hb and GSH	(1.5 ± 0.15) · 10^−4^
Figure 6	(**I**)	Interaction with Hb	(6.4 ± 0.6) · 10^−4^
Figure 7	(**I**)	Decomposition	(1.3 ± 0.1) · 10^−4^
Figure 8	(**I**)	Interaction with GSH	(6.9 ± 0.7) · 10^−5^

**Table 3:** The results of mass spectrometry (Figure 9).

Ion mass singly charged, *m/z *	Ion type	Formula for M, subunit or sequence	Origin and other comments
308	[M + H]^+^	GSH	Glutathione, C_10_O_6_N_3_SH_17_
477	[M − GSH − 2NO + H]^+^	[Fe_2_(GSH)_2_(NO)_4_]	Decomposition of (**II**)
538	[M − GSH + H]^+^	[Fe_2_(GSH)_2_(NO)_4_]	Decomposition of (**II**)
613	[M + H]^+^	GS-SG	Oxidized form of glutathione
845	[M + H]^+^	[Fe_2_(GSH)_2_(NO)_4_]	Cation of (**II**)
867	[M + Na]^+^	[Fe_2_(GSH)_2_(NO)_4_]	Cation of (**II**)-Na^+^

(**II**) is the complex [Fe_2_(SC_10_H_17_N_3_O_6_)_2_(NO)_4_]SO_4_·2H_2_O.

## Abbreviations

(**I**): Complex [Fe_2_(SC_5_H_11_NO_2_)_2_(NO)_4_]SO_4_·5H_2_O
(**II**): Vomplex [Fe_2_(SC_10_H_17_N_3_O_6_)_2_(NO)_4_]SO_4_·2H_2_O
NIC: Nitrosyl iron complex.
