# Supplementary material for: Reversible Dissociation and Ligand-Glutathione Exchange Reaction in Binuclear Cationic Tetranitrosyl Iron Complex with Penicillamine
Source: Bioinorg Chem Appl. 2014 Mar 25;2014:641407. doi: 10.1155/2014/641407 (PMC3984828; doi:10.1155/2014/641407)
Supplement: Supplementary file 1 — Kinetics of change of difference spectra at the interaction of ( I ) and ( II ) with Hb. ( I ) complex [Fe2(SC5H11NО 2)2(NO)4]SO4 ∙5H2O. ( II ) complex [Fe2(SC10H17N3O6)2(NO)4]SO4•2H2O). [file 641407.f1.pdf]

Supplementary data.

Kinetics of change of difference spectra at the interaction of ***(I)*** and ***(Ii)*** with Hb.

(I) complex $[Fe_2(SC_5H_{11}NO_2)_2(NO)_4]SO_4\cdot 5H_2O$

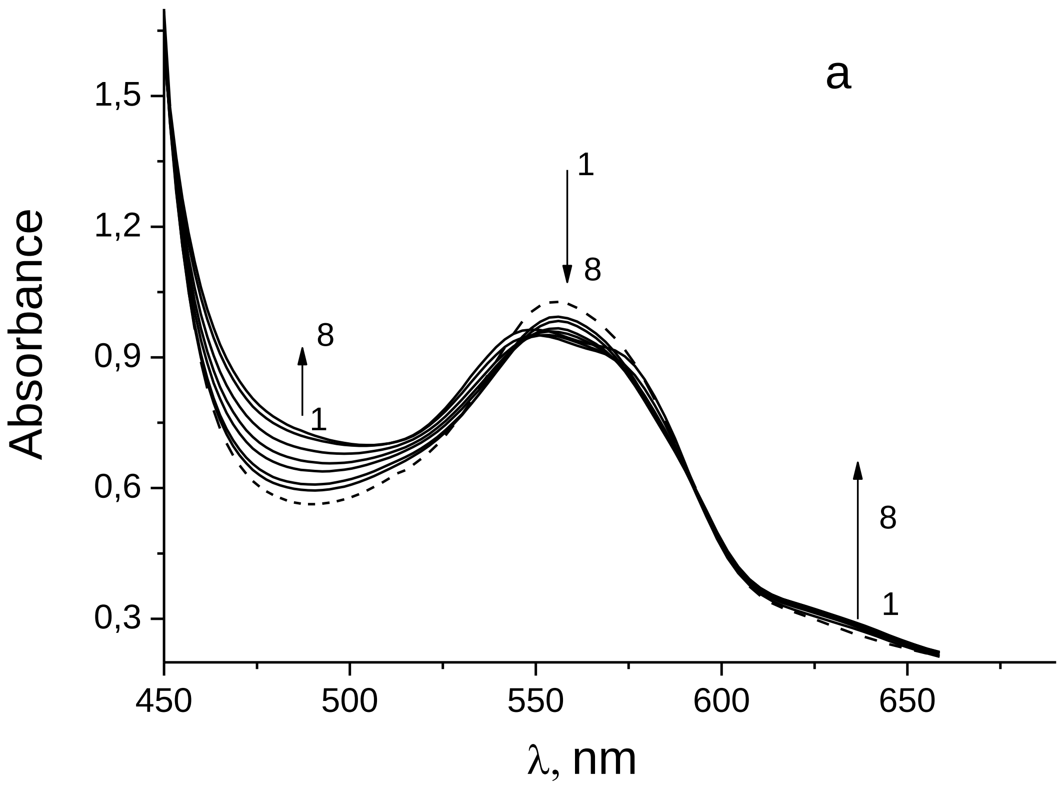

(II) complex $[Fe_2(SC_{10}H_{17}N_3O_6)_2(NO)_4]SO_4\cdot 2H_2O)$

a) Kinetics of change of difference spectra at the interaction of ***(Ii)*** ($1.5\cdot 10^{-4}$ M) with Hb ($2\cdot 10^{-5}$ M). Dotted line (1) is spectrum of Hb. Spectra 2-8 were registered at 0.5 (2), 1 (3), 3 (4), 5 (5), 7 (6), 9 (7), 11(8) h after start of reaction. Conditions of reaction: 25 ^{o}C, solvent is 0.05 M Tris-HCl buffer, pH 7.0.

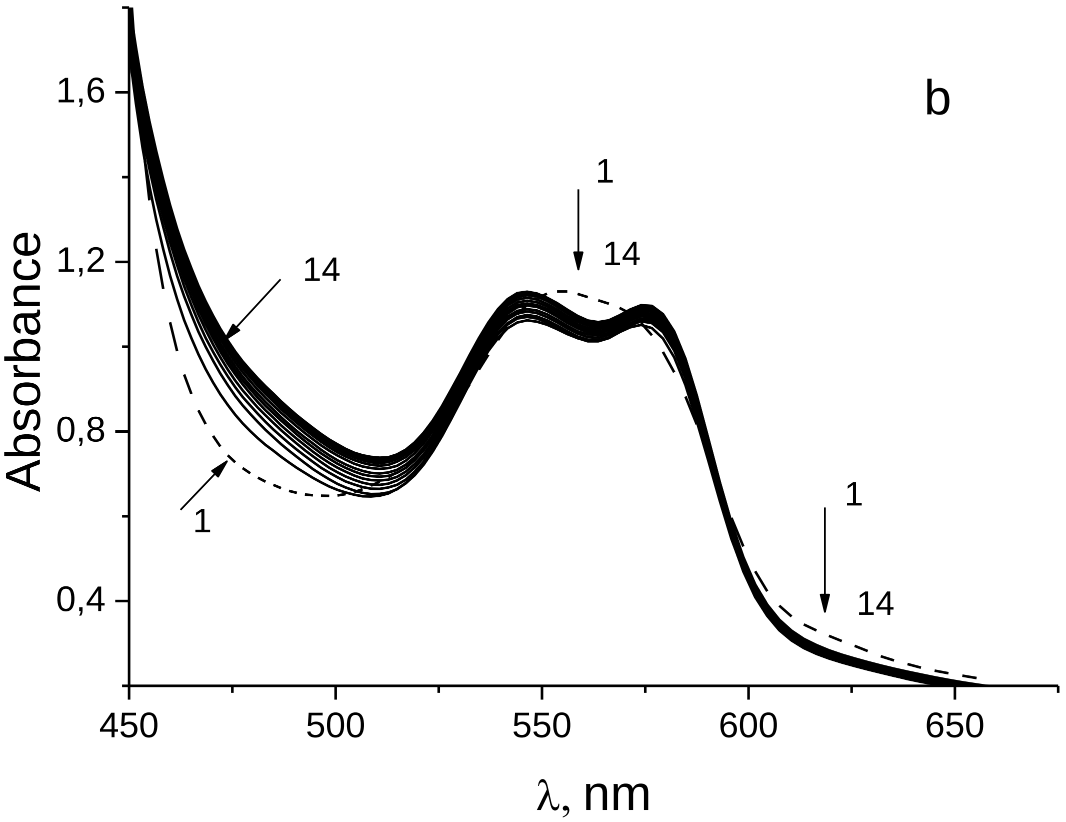

b) Kinetics of change of difference spectra at the interaction of ***(I)*** (1.5·10^{-4} M) with Hb (2·10^{-5} M). Dotted line (1) is the spectrum of Hb. Spectra 2-14 were registered at 0.05 (2), 0.16 (3), 0.33 (4), 0.5 (5), 0.66 (6), 0.8 (7), 1 (8), 1.25 (9), 1.5 (10), 1.75 (11) 2 (12), 2.23 (13) и 2.5 (14) h after start of reaction. Conditions of reaction: 25 ^{o}C, solvent is 0.05 M Tris-HCl buffer, pH 7.0

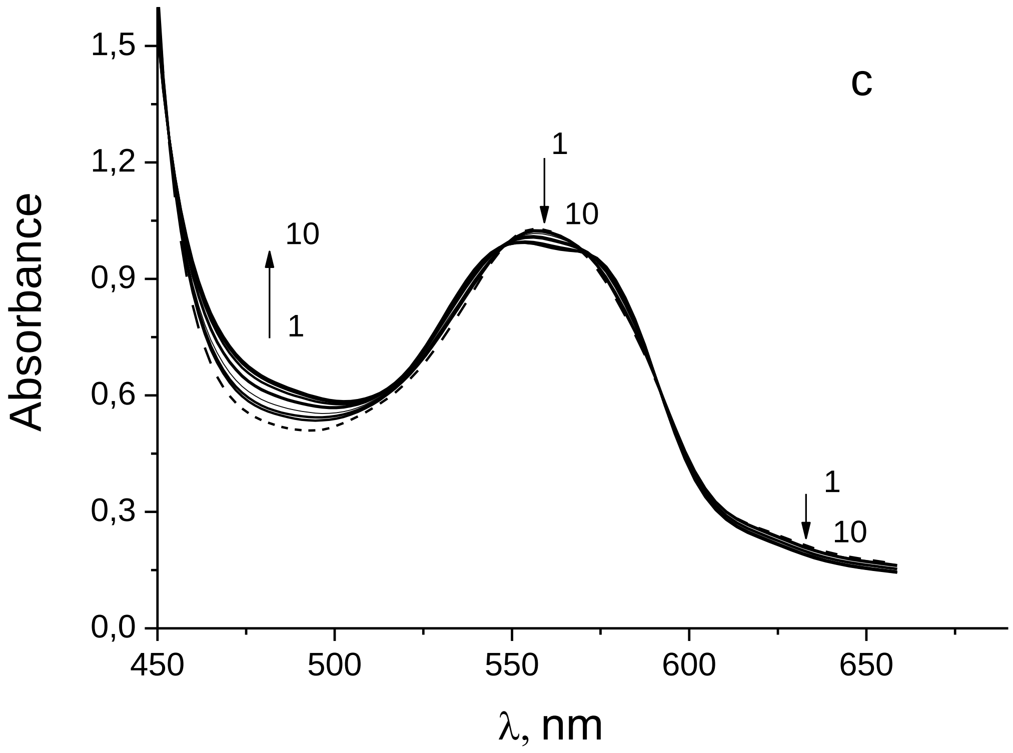

c) Kinetics of change of difference spectra at the interaction of ***(I)*** ($1.5 \cdot 10^{-4}$ M) with GSH (10^{-3} M) in Hb ($2 \cdot 10^{-5}$ M) presence. Dotted line (1) is the spectrum of Hb. Spectra 2-10 were registered at 0.01 (2), 0.16 (3), 0.3 (4), 1.4 (5), 2 (6), 3 (7), 4 (8), 4.5 (9), 5 (10) h after start of reaction. Conditions of reaction: 25 ^{o}C, solvent is 0.05 M Tris-HCl buffer, pH 7.0.
